# Classification Videos Reveal the Visual Information Driving Complex Real-World Speeded Decisions

**DOI:** 10.3389/fpsyg.2018.02229

**Published:** 2018-11-20

**Authors:** Sepehr Jalali, Sian E. Martin, Colm P. Murphy, Joshua A. Solomon, Kielan Yarrow

**Affiliations:** ^1^Department of Psychology, City, University of London, London, United Kingdom; ^2^Expert Performance and Skill Acquisition Research Group, School of Sport, Health and Applied Science, St Mary's University, Twickenham, United Kingdom; ^3^Centre for Applied Vision Science, City, University of London, London, United Kingdom

**Keywords:** reverse correlation, classification images, sports science, visual perception, tennis, occlusion, bubbles, spatiotemporal

## Abstract

Humans can rapidly discriminate complex scenarios as they unfold in real time, for example during law enforcement or, more prosaically, driving and sport. Such decision-making improves with experience, as new sources of information are exploited. For example, sports experts are able to predict the outcome of their opponent's next action (e.g., a tennis stroke) based on kinematic cues “read” from preparatory body movements. Here, we explore the use of psychophysical classification-image techniques to reveal how participants interpret complex scenarios. We used sport as a test case, filming tennis players serving and hitting ground strokes, each with two possible directions. These videos were presented to novices and club-level amateurs, running from 0.8 s before to 0.2 s after racquet-ball contact. During practice, participants anticipated shot direction under a time limit targeting 90% accuracy. Participants then viewed videos through Gaussian windows (“bubbles”) placed at random in the temporal, spatial or spatiotemporal domains. Comparing bubbles from correct and incorrect trials revealed how information from different regions contributed toward a correct response. Temporally, only later frames of the videos supported accurate responding (from ~0.05 s before ball contact to 0.1 s afterwards). Spatially, information was accrued from the ball's trajectory and from the opponent's head. Spatiotemporal bubbles again highlighted ball trajectory information, but seemed susceptible to an attentional cuing artifact, which may caution against their wider use. Overall, bubbles proved effective in revealing regions of information accrual, and could thus be applied to help understand choice behavior in a range of ecologically valid situations.

## Introduction

Imagine yourself driving your car one evening. As you turn a bend, a cat appears in your headlights. Should you brake hard, or perhaps swerve left or right? Seemingly without your conscious intervention, your body has decided, and you are relieved to find that your reaction has avoided the cat without causing a more dangerous collision.

Successful speeded decision-making of this kind has been fundamental to our survival as a species, and continues to pervade everyday life. However, it is not always obvious what particular information is exploited to make speeded choices, and which potentially relevant cues are left unused. For example, when avoiding the cat, was the upcoming curvature of the road or the presence of another vehicle in the rear-view mirror taken into account? If not, might a better driver have exploited these cues?

In real-life scenarios, many cues to speeded decision-making are subtle, and training or extensive experience may be required to facilitate their use. Competitive sport provides a good example. How is it that experts are able to quickly and accurately discriminate sporting scenarios as they unfold? Previous research has revealed that elite athletes make use of visual information from their opponents' bodies in order to predict what will happen next, for example using the movement of a cricket bowler's arm and hand, just before ball release, to anticipate the trajectory of the ball that will be delivered (Abernethy and Russell, 1984; Muller et al., 2006; Yarrow et al., 2009).

Our knowledge about this sport's “expert anticipatory advantage” has been garnered through the application of the spatial and temporal occlusion paradigms, developed by experimental psychologists (e.g., Jones and Miles, 1978; Abernethy, 1988). However, there are several issues with these paradigms as a general-purpose methodology to reveal regions of information accrual in complex real-world scenarios. In the next section, we briefly describe these traditional approaches, then use their limitations to motivate the introduction of a method that has thus far been applied mainly to low-level psychophysical problems: Classification-image analysis (Ahumada and Lovell, 1971). We go on to describe one specific variant of this approach (“bubbles;” Gosselin and Schyns, 2001) which we will test here, using tennis as a representative decision-making scenario, in order to assess its applicability to the more general problem of measuring information extraction in complex situations where one from a discrete set of choices must be rapidly selected.

### The spatial and temporal occlusion paradigms

In competitive sports, time is of the essence. While an unfolding scenario might ultimately provide unambiguous information about the appropriate response, this will often come too late for an athlete to simply wait and then react with certainty. Examples include reacting to bowling in cricket, pitching in baseball, serving in tennis, or penalty taking in soccer. In each case, the ball's trajectory provides the clearest information about the appropriate reaction, but the interval of time between receiving this information and having to initiate a response is very brief. This necessitates some degree of guessing if the ball is to be intercepted effectively. However, this guessing may still be informed by additional cues, for example the kinematics of the opponent's body prior to ball contact or release. To investigate this issue, multiple exemplars of a sports scenario can be filmed from a decision maker's perspective—for example, tennis serves coming to either forehand or backhand—so that a realistic decision with *n* (in this case 2) possible responses can be elicited. The videos can then be deliberately degraded, under the logic that the decision, which is trivially easy when the video is played in its entirety, will become much harder as critical cues are removed (ultimately falling to chance levels of performance).

Early studies degraded videos by limiting information in the temporal domain, known as temporal occlusion. For example, in tennis (the sport we investigate here) one early study showed that experts were above chance (and better than intermediate or novice players) at guessing the landing position of a serve when the video was stopped at (and thus information was occluded from) 0.042 s before ball contact (Jones and Miles, 1978). The implication was that some useful information must have been accrued before this moment. Typically, temporal occlusion involves stopping the video at one or several different time points, but some authors have also introduced discrete windows (e.g., 0.3 s periods of visibility) that occlude both earlier and later information (e.g., Farrow et al., 2005).

Temporal occlusion approaches can be complemented by spatial occlusion, where the video is shown after having removed a spatially constrained source of information, in order to assess its impact. In tennis, this is typically accompanied by full (temporal) occlusion following racquet-ball contact in order to isolate the spatial location of cues utilized for *pre-trajectory* prediction. For example, Jackson and Mogan (2007) showed that experts still discriminated the direction of tennis serves at above-chance levels following removal of body regions such as the entire lower body, but not when the ball's toss was occluded. Experts were also impaired (but to a lesser extent) by removal of the arm and racquet. Removal of this latter region has also been found to impair expert performance when predicting the direction of ground strokes, rather than serves (Shim et al., 2006).

The temporal and spatial occlusion approaches have provided important information about how experts extract and use information in numerous sporting domains. In principal the approaches could even be generalized beyond sporting scenarios. However, they have some drawbacks as widely applicable methods. First, they depend upon the researcher's intuitions regarding the location of relevant information—the researcher is choosing what to occlude. It may be desirable to have sources of information emerge in a more bottom-up fashion, to make sure that cues are not overlooked (and avoid concerns over experimenter confirmation bias). Second, the creation of stimuli is time intensive. Video manipulation of this kind, particularly for spatial occlusion, is difficult to automate, providing a barrier to potential users from new fields of experimentation.

Spatial and temporal occlusion techniques were developed by researchers in applied cognitive psychology. However, as we outline next, parallel developments in other fields, most notably sensory psychophysics, provide a natural complement to these techniques that relies on a very similar basic logic, but replaces deliberate image occlusion with *random* degradation.

### Classification-image techniques

Traditional psychophysics (e.g., Graham, 1989) has three general paradigms for probing the properties of visual mechanisms: summation, masking, and adaptation. All three paradigms require a visual *target* that observers can detect. In *m*-alternative, forced-choice designs, where there is 1 target and *m*−1 *foils*, non-target stimuli added to the target typically produce a decrease in the detection threshold (i.e., less of the target is required for successful detection). This is known as summation. Selectivity of the detection mechanism can be inferred from the relationship between non-target content and threshold decrease. In the masking paradigm, non-target stimuli are added to all *m* alternatives. This typically (but not always) elevates detection threshold, and selectivity of the detection mechanism can be inferred from the relationship between non-target content and threshold elevation. The adaptation paradigm is like masking, except the non-target stimuli are presented prior to the *m* alternatives.

Unlike *m-*alternative designs, each trial in a *classification* design contains only 1 target (there are no foils). The observer must classify this stimulus into one of *n* possible categories (note the similarity to the occlusion paradigms described previously). With only a target (and no foils) there is no difference between masking and summation. Non-target stimuli added to the target can bias the observer's response and/or reduce its reliability. In a typical experiment, non-target content is manipulated systematically, and its effect on response bias and response reliability can provide clues to the observer's decision process.

Instead of manipulating non-target content systematically, Ahumada et al. (Ahumada and Lovell, 1971; Ahumada, 2002) pioneered the use of *stochastic* manipulation. In their studies, the selectivity of classification mechanisms was inferred from the trial-by-trial relationship between each individual sample of the non-target or “mask” and the observer's response. In some cases (e.g., Abbey et al., 1999) a simple linear combination of non-target stimuli (called the “classification image”) could be guaranteed to provide an unbiased estimate of the classifier's “template” or receptive field. Essentially, the random noise that happened to be added to the image when observers got things right (and indeed the random noise added when they got things wrong) can be extremely informative about how they are forming their decisions.

**Figure 1:**
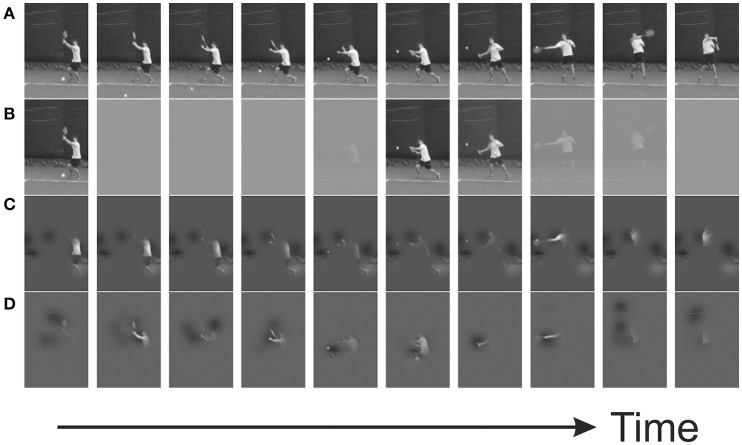
Example trial from a bubbles experiment, in which Gaussian profiled windows of visibility are placed at random positions. **(A)** Original video sequence; **(B)** temporal bubbles, revealing information only at specific times; **(C)** spatial bubbles, revealing information only in specific positions; **(D)** spatiotemporal bubbles—spatially constrained regions of information have limited lifetimes.

The traditional classification-image approach in visual psychophysics makes use of pixel-by-pixel additive luminance noise, and is conceptually closely related to the technique of spike-triggered averaging applied to single-cell recordings in neurophysiology (Marmarelis and Naka, 1972; Simoncelli et al., 2004). It is sometimes referred to as “reverse correlation,” and can appear mathematically intimidating to the uninitiated. However, a closely related approach, based on the stochastic application of multiplicative noise, is (arguably) more intuitive. In the “bubbles” approach, the entire information space (e.g., a 2D image) is initially masked (e.g., set to average image luminance) before specific regions are revealed through randomly located Gaussian windows (the so-called bubbles) that vary from trial to trial (see Figure 1 for illustration). As we expand in the methods section below, a comparison of the bubbles that were present on trials where participants succeeded with those present on trials where they failed can be used to produce a classification image yielding a map of the informative regions driving correct decisions. For example, bubbles have been used to show which regions of the human face are used by observers when they make decisions about gender (Gosselin and Schyns, 2001).

### The current study: testing bubbles for real-world decisions

The bubbles technique has previously been applied mainly to static images, although bubbles with temporal or spatiotemporal profiles have sometimes been applied in order to reveal information use through time (e.g., Vinette et al., 2004; Fiset et al., 2009; Blais et al., 2013). Occasionally, dynamic stimuli more akin to a video have been investigated (e.g., Thurman and Grossman, 2008; Blais et al., 2012). However, given the psychophysical tradition within which classification-image analysis evolved, the tendency has been to work with austere and tightly controlled stimuli. Here, we investigate the use of bubbles to reveal informative regions within real-world video stimuli. We also apply different bubbling methods (temporal, spatial, and spatiotemporal) to the same task to see how each performs. Furthermore, we deliberately adopt a sample size and experimental duration typical of experimental psychology, rather than sensory psychophysics, as classification-image approaches have tended to be used with small samples but very large numbers of trials (but see e.g., Butler et al., 2010; Smith et al., 2017), something that may appear as a barrier to researchers with a more applied focus (who may depend on specialist populations). We use sports, specifically tennis, as a test case, with the intention of assessing the applicability of this kind of approach to a wider range of decision-making scenarios.

## Methods

### Participants

Thirty participants (7 women and 23 men) aged 19–62 (mean = 32) took part in the various stages of this experiment (with 29 participants completing each of the stages, and most participants completing all three). Participants were recruited and assigned to one of two groups on the basis of their tennis playing experience/skill. Those in the novice group (5 women and 10 men) aged 20–51 years (mean = 30) had no experience of playing tennis competitively. Those in the tennis group (2 women and 13 men) aged 19–62 years (mean = 33) had 2–35 (mean = 11) years of experience playing competitive tennis and currently played between 0 and 150 (mean = 30) competitive matches per year^1^. Players also indicated their current International Tennis Number (ITN), which is an index of their standard of play and ranges from ITN 1 (a player with extensive professional tournament experience and who currently holds or is capable of holding an ATP/WTA ranking) to ITN 10 (a player that is just starting to play competitively). Tennis-playing participants had an average ITN of 4 (range 2–7). Informed consent was obtained from all participants, who were paid £10/h for their time. Ethical approval was granted by the Dept. of Psychology Research Ethics Committee, City, University of London.

### Apparatus and stimuli

Video stimuli (available on request) were recorded at a tennis club using a tripod-mounted camera (frame rate 120 Hz, frame size 1,280 × 720 pixels). Four club coaches/hitters of a good but not elite standard acted as models, and were instructed to “hit winners” without attempting explicit deception. They were situated near the baseline, and recorded against a largely uniform blue backdrop. They were recorded serving (from the right-hand side of the court) or playing forehand ground strokes (running rightwards from a central position to return near the singles side line), directing their shots toward an imaginary receiver's forehand or backhand. To increase image resolution, the camera was positioned at the net, on a line projecting from the filmed player to the imaginary receiver at the opposite baseline (height = 1.6 m, left of center line by 1.25 m for ground strokes, right of center line by 1.5 m for serves). Balls were called in or out to facilitate later rejection of videos where the ball landed out. For ground strokes, one player delivered to all of the other three models, to ensure as constant a delivery as possible, and also called for line/cross strokes (i.e., toward the right-handed model's backhand and forehand, respectively) immediately after delivery to prevent early decisions that might introduce unnatural or pre-emptive postural cues. Only these three models were included in the experimental trials (see below). The final player received deliveries from a different model, and was consequently included only in practice trials.

Videos were first transformed to eight-bit gray scale. Of 350 initial videos, 215 contained shots that landed in. These videos were retained and then rated by two authors in order to pick a subset that were unambiguous (regarding the direction of the shot—line/cross for ground strokes, T/cross for serves), relatively homogeneous in terms of the position of the players at the time of ball contact, and lacking in artifactual cues that might allow the videos to be easily remembered for future classification (e.g., an unusual delivery trajectory for ground strokes). In each video, the frame corresponding to ball contact and the position at which the ball struck the racquet head on this frame were manually identified for use in subsequent presentation and analysis (see below).

The experiment was controlled by a PC running scripts written in Matlab (The Mathworks, Natick, U.S.A.) using the Psychophysics Toolbox extension (Brainard, 1997; Pelli, 1997; Kleiner et al., 2007). Video stimuli were presented on a CRT monitor (1,024 × 768 pixels, ~40 × 30 cm, with a vertical refresh rate of 120 Hz). Only a central 600 × 400 pixel region of each video that excluded irrelevant peripheral information was presented. The screen was elevated to eye level via an adjustable support and viewed at a distance of ~100 cm in order to present the opposing tennis player with a height subtending ~4° visual angle (approximating their size as seen from the baseline during actual play). Participants responded by stepping rightward or leftward, thus lifting the corresponding foot from one of two digital pedals, monitored at 100,000 Hz via a 16 bit A/D card (National Instruments X-series PCIe-6323).

### Design and procedure

Participants completed three variants of the task in separate sessions, with a constant order (temporal, then spatial, then spatiotemporal)^2^. Sessions took around 2 h, and consisted of four blocks: One practice and one experimental block presenting videos of only serves, and the same for ground strokes (with order of shot type counterbalanced across participants). During practice, participants viewed 100 videos (50% to forehand, 50% to backhand) containing all four players (8 possible videos per player) but with a preponderance of videos (70%) from one player (see stimuli, above) and fewer videos (10% each) from the remaining three players, who were saved mainly for the experimental trials (see below). Videos were presented in a random order, and selection was carried out with replacement (such that individual videos for each player did not necessarily occur with equal frequencies).

Videos presentations began at −0.8 s relative to racquet-ball contact, and terminated at 0.2 s after racquet-ball contact, or at the time of response if earlier than this. We wished to push participants to respond as quickly as was feasible for them, while retaining some ability to perform the task, so as to extract sources of information that might be used during actual play. The practice block therefore served not only as a warm up, but also to estimate the time window within which participants could respond with ~90% accuracy. This was achieved via a quest staircase (Watson and Pelli, 1983) modified to assume a cumulative Gaussian psychometric function. An adjustable value defined the middle of a 0.3 s window within which participants were encouraged to respond via on-screen feedback (which also indicated correctness and the exact time they took to act). Quest varied this value, based on the correctness of previous decisions (but only those decisions that had been made within the target window) in order to estimate an appropriate response deadline for the subsequent experimental block (being the upper limit of the target window). The initial target value was 0.4 s from racquet-ball contact. Further Quest parameters, in particular the slope of the assumed psychometric function (σ^−1^ = 7.5 s^−1^) were estimated from pilot work, in which the target window for one author was manipulated systematically, via the method of constant stimuli.

For the experimental blocks, 24 new videos (8 per player, 50% to forehand and 50% to backhand) were selected from the three players seen less often during practice. These videos were presented 16 times each in a random order, yielding a block of 384 trials. Participants were required to respond by their previously established deadline, and trials where they failed to do so (along with any trials with presentation glitches, i.e., where one or more frames were dropped after the −0.2 s time point) were re-randomized and repeated at the end of the block. Feedback about response times and correctness was provided after every trial.

Importantly, during experimental trials, the videos were subjected to random masking via the application of bubbles (see Figure 1, and Videos S1–S3). In different sessions, individual bubbles were combined to generate bubbles profiles in one (temporal), two (spatial) or three (spatiotemporal) dimensions. The number of bubbles presented (*B*) began at 12. This number was then adjusted (up to ceiling values of 20, 20, and 90 for temporal, spatial, and spatiotemporal sessions, respectively) via a Quest staircase varying the number of bubbles in order to maintain participants' performance at around 75% correct (i.e., bubbles were added if the task was too hard, or removed if it was too easy). The profile of each individual bubble was that of a 1, 2, or 3-dimensional Gaussian density function, scaled to have unit height. In the temporal sessions its width (σ) was 3 frames; in the spatial sessions its width was 12 pixels (vertically and horizontally); and in the spatiotemporal sessions its widths were 5 frames and 12 pixels^3^.

Bubble mean positions were generally selected at random within a domain extending throughout the relevant space of the video. However, in the spatiotemporal session, mean bubble positions were excluded from the first 25 frames of the video, and were further constrained to a rectangular spatial region of the video that varied across frames, capturing all player motion, in order to generate fewer bubbles in regions of null information^4^. Bubbles profiles were determined by combining the individual bubbles together. This was achieved by first reflecting bubble magnitudes around 0.5, then multiplying them together, and finally re-reflecting:

(1) $$\begin{array}{r} \text{Bubbles}=1-\;\prod_{b=1}^{B}\left(1-{\text{bubble}}_{b}\right) \end{array}$$

Pixel intensities were then calculated for display as the mean pixel intensity plus the difference between original and mean intensities (at each point) multiplied by the Bubbles profile (at that same point). Expressed in terms of Weber contrasts, pixels were displayed at their original Weber contrasts multiplied by the Bubbles profile.

### Data analysis

The saved Bubbles profiles from each trial formed the starting point in generating classification sequences, images, or videos (for temporal, spatial, and spatiotemporal sessions, respectively), which reveal the regions from which information supporting a correct response has been extracted. We collectively term these *classification arrays*. First, for spatial and spatiotemporal sessions only, Bubbles were re-centered so that the profile (saved in video coordinates) was translated to a new coordinate frame centered on the ball at the time of racquet-ball contact. This has the effect of reducing noise in subsequent estimation, but to a degree that depends upon the proximity of any potential region of information to the middle of the new coordinate frame^5^. Essentially, it addresses the problem that when multiple videos are used, it is not necessarily absolute spatial position that matters—it might, for example, be the position of a body part, which is best captured by a body-centered frame of reference.

Next, for each participant, a weighted sum of (re-centered) Bubbles profiles (weighting profiles from correct trials positively and profiles from incorrect trials negatively) yielded the raw classification array:

(2) $$\begin{array}{r} \text{RCA}=\;\sum_{c=1}^{C}{\text{Bubbles}}_{c}\;-\sum_{i=1}^{I}{\text{Bubbles}}_{i} \end{array}$$

However, in order to provide more intuitive values for visualizing and combining data across participants (and to make the method generalizable to cases where different participants completed different numbers of trials) raw classification arrays were normalized to a *z*-like format. This was achieved via a permutation approach. On each of 2,000 iterations, correct/incorrect labels were randomly re-assigned (without replacement) to individual trials. The means and standard deviations at each point (i.e., each frame and/or pixel) calculated over these 2,000 permutations were used to *z*-score the classification array. This yielded an array varying about zero, with positive values indicating regions of possible information accrual.

In order to draw statistical inferences across large arrays while controlling familywise type 1 error appropriately, data from all participants were combined and assessed via both cluster and t_max_ (also known as pixel or single-threshold) corrected permutation tests (Blair and Karniski, 1993; Nichols and Holmes, 2002; Groppe et al., 2011). The first step for both tests was to transform the *z*-scores at each point into a one-sample *t* statistic (i.e., the ratio of the mean to the standard error across observers). For the t_max_ test, each of these *t* statistics was then compared with a “null” distribution of t_max_, the calculation of which is described below. Individual values of *t* greater than the 95th percentile of this null distribution were deemed significant, according to the t_max_ test. Under the null hypothesis, *t* scores should fluctuate randomly around zero. Permutation tests rely upon the construction of a null distribution consistent with the null hypothesis. Hence, prior to computing each value of t_max_ for the null distribution, the *z*-transformed classification array from each observer was multiplied by −1 with probability 0.5. A new *t* statistic (summarizing the results from all participants) was then computed for each point in the array. The maximum (across points) of these values (unsigned) is deemed t_max_. For our t_max_ test, we used a null distribution of 1,999 values computed in this manner.

For the cluster test, a cluster was defined as the sum of contiguous *t* values where *t* exceeded an (arbitrary) 5% threshold (two-tailed). Note that neither the particular way in which a cluster is defined, nor the particular threshold that defines inclusion in a cluster, affect the logic by which the procedure yields control over type 1 errors (so long as multiple definitions and/or thresholds are not tried out in order to cherry pick a preferred result). Contiguity was defined as adjacent frames in the 1D case. In the 2D case it was defined as 4-connected^6^ pixels. Finally, in the 3D case it was defined as 4-connected pixels per frame, but only the largest cluster across *all* frames of the video was used to form the null distribution^7^. Clusters whose summed *t* values exceeded the 95th percentile in a null distribution of cluster sums were deemed significant. Sums for the null distribution were computed in a manner analogous to the computation of t_max_, i.e., following a random reassignment of sign: the random multiplication of each observer's *z*-transformed classification array by −1 with probability 0.5. Just like the null distributions of t_max_, our null distributions of cluster sums were formed from 1999 recomputations of *t* following this random reassignment of sign.

Subsets of trials forming repeated-measures comparisons (e.g., information accrued from shots to forehand vs. shots to backhand) were compared by subjecting *differences* of classification arrays to the procedure outlined above. For comparisons between groups (e.g., tennis players vs. novices) the same procedure was followed, with modifications following standard principles for permutation testing (i.e., group labels were randomly shuffled on each permutation). Matlab code for our experiments and analyses are available at http://www.hexicon.co.uk/Kielan/#research.

## Results

### Display characteristics and response times

Response deadlines where imposed in experimental sessions, based on performance during practice, in order to ensure that participants used the earliest information source available to them. Deadlines in each group, experiment and condition are shown in Table 1, along with mean RTs on accepted trials (which are necessarily lower than the deadlines). Table 1 also shows mean accuracy and mean number of bubbles during experimental blocks. Novices and tennis players differed significantly on only one of these metrics [mean RT was lower for tennis players than novices in the ground-strokes trials of the spatiotemporal experiment: independent *t*_(28)_ = 2.451, *p* = 0.021]. However, given the familywise context (i.e., 24 such tests) the Dunn-Šidák corrected *p*-value was not significant (*p* = 0.395).

**Table 1:** Mean (standard deviation) of response deadlines, reaction times (RT), accuracy, and number of bubbles for novices and experts responding to ground strokes (G.S.) and serves in temporal, spatial, and spatiotemporal experiments.

		**Novices**				**Tennis players**			
		**Deadline (s)**	**RT** **(s)**	**Correct (%)**	**Bubbles (N)**	**Deadline (s)**	**RT** **(s)**	**Correct (%)**	**Bubbles (N)**
Temporal	G.S.	0.40 (0.08)	0.24 (0.05)	69 (5)	12 (5)	0.36 (0.07)	0.20 (0.05)	68 (4)	11 (5)
	Serves	0.43 (0.08)	0.25 (0.05)	69 (5)	11 (5)	0.43 (0.07)	0.23 (0.08)	71 (6)	10 (4)
Spatial	G.S.	0.42 (0.09)	0.25 (0.11)	66 (7)	14 (4)	0.42 (0.06)	0.26 (0.04)	68 (3)	13 (3)
	Serves	0.45 (0.08)	0.27 (0.08)	68 (6)	13 (6)	0.47 (0.06)	0.28 (0.04)	70 (3)	13 (4)
Spatio- temporal	G.S.	0.43 (0.08)	0.29 (0.06)	66 (6)	59 (22)	0.38 (0.06)	0.22 (0.09)	62 (9)	61 (24)
	Serves	0.50 (0.09)	0.30 (0.08)	60 (7)	79 (10)	0.46 (0.09)	0.24 (0.08)	59 (7)	77 (11)

*Response deadlines and reaction times are relative to the point of racquet-ball contact*.

Although our Quest staircase aimed to generate 75% performance, the somewhat lower accuracy scores are likely the result of the caps we imposed on the maximum number of bubbles, in combination with the response deadline. Nonetheless, performance was above chance in all conditions, implying scope for bubbles to reveal the sources of information that were informing correct decisions.

### Temporal bubbles: informative regions

**Figure 2:**
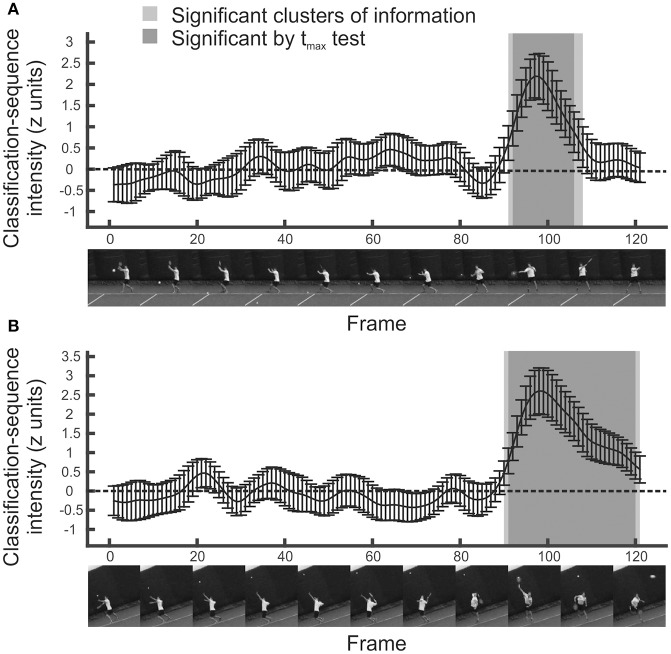
Mean classification sequences for all participants in temporal bubbles experiments. **(A)** Ground strokes. **(B)** Serves. Shaded regions were significant in cluster/t_max_ permutation testing, suggesting information was extracted from this part of the video sequence. Error bars denote 95% confidence intervals around classification arrays.

The mean *z*-scored classification arrays (for the entire sample) for the temporal experiment are shown in Figure 2. Positive values indicate video frames that are candidates for periods of information extraction. For the ground strokes, two regions are promising. The most obvious one extends from around frame 90 (so ~0.050 s before racquet-ball contact) until around frame 108 (so ~0.1 s after racquet-ball contact). A much smaller region of positivity occurs around frame 64 (~0.267 s before racquet-ball contact, when the swing is being initiated).

The statistical significance of these regions was assessed using cluster and t_max_ permutation tests. t_max_ tests are well suited for detecting strong and highly localized regions of information, while cluster tests are well suited for detecting more diffuse regions (Chauvin et al., 2005). Both control familywise error across a classification array, but cluster tests do not guarantee strong familywise error rate control at *every* constituent point (Nichols and Holmes, 2002; Groppe et al., 2011). The permutation approach avoids strong distributional assumptions. It revealed that only the latter putative information-carrying region represented a significant cluster (extending from frame 91 to frame 108; *p* = 0.0005). Note, however, that the bubbles technique introduces smear (dependent on the extent of the individual bubbles) such that the recovered classification array should be considered a filtered approximation of the information it attempts to represent. Hence we can conclude that information was extracted somewhere within this temporal region, but should not infer that each and every one of these frames provided useful information for the classification of shot direction, even for those significant by t_max_ test. We revisit and expand upon this issue (via a set of simulations) in the final section of the results.

Analyzing responses to the serve stimuli generated a similar result (Figure 2, bottom). While there is a suggestion of information accrual early on during the ball toss, around frame 20, only the large and striking region from frame 90 onwards forms a significant cluster (*p* = 0.0005). From these data, we can conclude that participants were basing their decisions on information presented late on in the videos, most likely from after the ball had been struck, but perhaps also from slightly before this point.

### Temporal bubbles: regions of contrast

**Figure 3:**
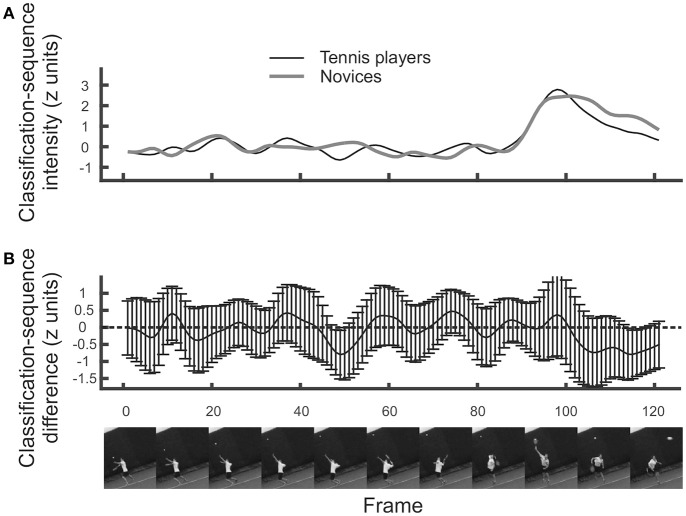
**(A)** Mean classification sequences shown separately for tennis players and novice groups in the temporal bubbles experiment involving serves. **(B)** Mean difference in classification sequences between the two groups. No significant differences emerged. Error bars denote 95% confidence intervals around classification arrays.

Just as with other forms of data, we can perform contrasts on classification arrays to determine whether particular regions are utilized more in one condition than in another. For the temporal data, we present an example of a between-participants contrast, by comparing the tennis-playing participants to the novices when responding to videos of serves. Results are illustrated in Figure 3. It is apparent that, slightly surprisingly, classification sequences are very similar between tennis players and novices (Figure 3, top)^8^. There is perhaps a suggestion that novices make slightly more use of ball trajectory information toward the very end of the videos, but this difference is not significant by cluster or t_max_ test (Figure 3, bottom).

### Spatial bubbles: informative regions

**Figure 4:**
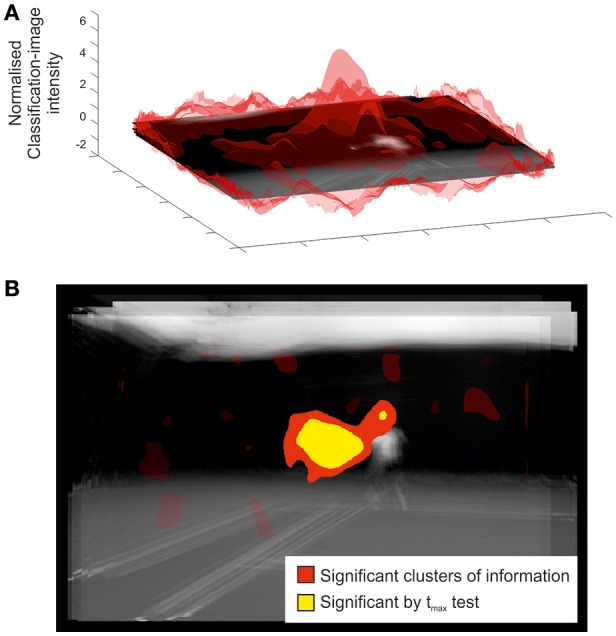
Classification image for all participants in the spatial bubbles experiment involving ground strokes. Results are overlaid on an image of the mean of all presented videos for the frames capturing racquet-ball contact, centered on the point of racquet-ball contact (hence constituent images do not perfectly align). However, the results of the spatial analysis are not specific to any one time point. **(A)** Transparent red (gray) peaks denote mean classification-image intensity normalized to the cluster threshold value used in permutation testing (i.e., values more extreme than ±1 formed potential clusters). **(B)** Solid colored regions were significant in cluster/t_max_ permutation testing, suggesting information was extracted from this part of the video. Transparent red (gray) regions denote non-significant clusters.

Figure 4 illustrates the classification image and inferential statistical results emerging from the spatial experiment. For concision, we present data from only the ground-stroke session, but the services session yielded a broadly similar outcome. The classification image is shown at the top of the figure, and implies a region centered roughly over the racquet head from which useful information may be being extracted. This is clearer in the bottom part of the figure, where statistical thresholding has been applied to produce a 2D representation. The cluster is highly significant (*p* = 0.005) and covers the region occupied by the racquet, arm, and head at the time of racquet-ball contact. As with the temporal results, smear generated by the experimental and analytical techniques means that we should be cautious about inferring that information has been extracted from all points within a significant cluster. The spatial analysis also tells us nothing about the time at which information was extracted from within this cluster. However, in concert with the relevant temporal results (Figure 1, top) it seems likely that the significant spatial cluster may be capturing primarily the early trajectory of the ball as it leaves the racquet head. However, the fact that it extends to the player's head region suggests that the models in our video may have followed the ball with their eyes/heads after hitting it, providing another potential cue for our participants to exploit when guessing shot direction.

### Spatial bubbles: regions of contrast

**Figure 5:**
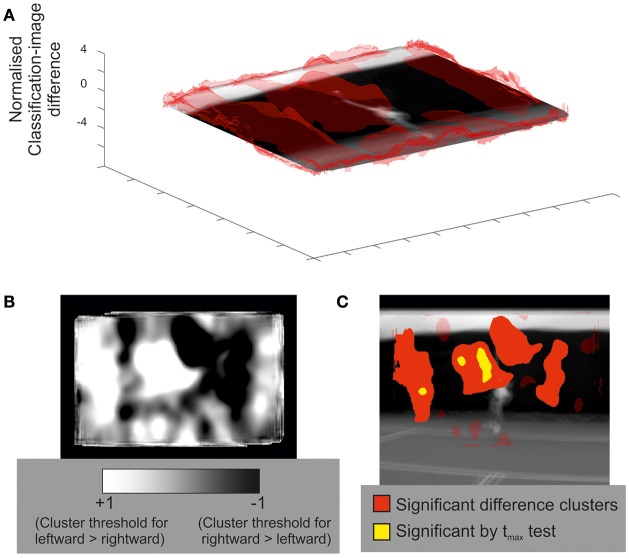
An illustrative within-participants contrast of classification images (rightward serves to forehand vs. leftward serves to backhand) for all participants in the spatial bubbles experiment. **(A)** Transparent red (gray) peaks denote mean classification-image differences, normalized to the cluster threshold value used in permutation testing (i.e., values more extreme than ±1 formed potential clusters). Results are overlaid on an image of the mean of all presented videos for the frames capturing racquet-ball contact, centered on the point of racquet-ball contact. **(B)** An alternative illustration of mean classification-image differences, normalized (as per part A) but trimmed at ±1 (the cluster threshold) and presented in 2D to better illustrate both positive and negative differences between conditions. **(C)** Solid-colored regions were significant in cluster/t_max_ permutation testing, suggesting that these parts of the video where more informative for one direction of shot than for the other. Compare with part B to ascertain the direction of the differences. Transparent red (gray) regions denote non-significant clusters.

Previously, for the temporal experiments, we presented an example of a between-participants contrast of classification sequences. It is also possible to run within-participant contrasts on the data from bubbles experiments. For example, we might ask whether different regions of the video drove decisions when the ball was delivered to forehand (on one half of all trials) compared to when it was delivered to backhand (on the other half). The results of this contrast are shown in Figure 5 for the spatial experiment involving predictions about service direction.

For contrasts of this kind, both directions of difference are potentially interesting, but a 3D visualization (Figure 5 part A) is better suited to illustrate one direction at a time (in this case leftward shots > rightwards shots). The heat plot in Figure 5 part B captures both directions of difference well, but it is difficult to see where, on the video, these differences lie. Figure 5 part C is complementary to parts A and B, but statistical thresholding has been applied, with clusters of significant difference overlaid on an averaged video frame. Together, the various visualizations show how regions to the left of the video, covering positions the ball might initially traverse when being hit toward a right hander's backhand, were more informative for exactly the subset of trials in which that stroke occurred (and vice versa for regions to the right of the video). From left to right, the four clusters are significant at *p* = 0.0065, *p* = 0.0045, *p* = 0.0045, and *p* = 0.039, respectively.

### Spatiotemporal bubbles

**Figure 6:**
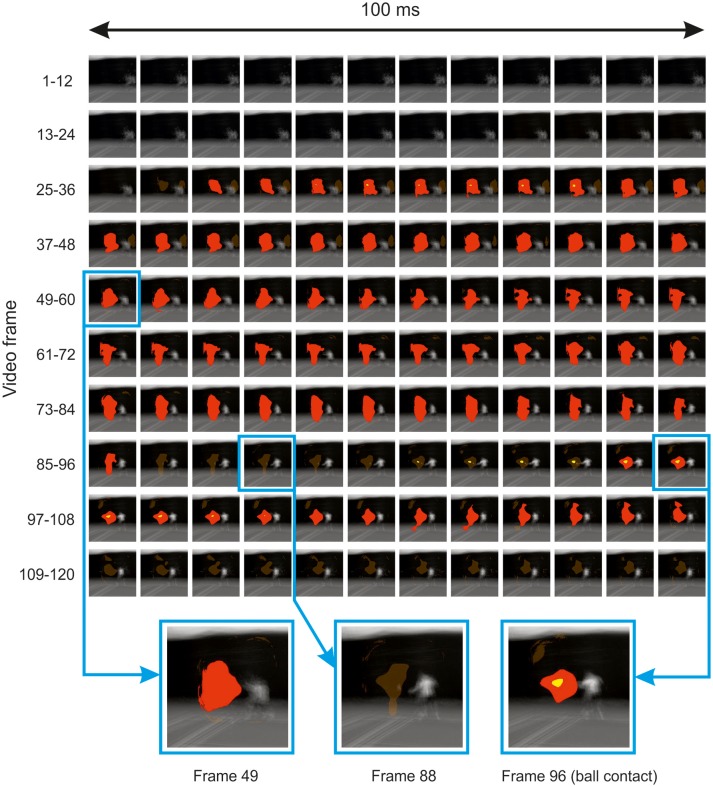
Thresholded classification video for all participants in the spatiotemporal bubbles experiment involving ground strokes. Results are overlaid on the mean of all presented videos (for each frame) centered on the point of racquet-ball contact (which occurred in frame 96). Solid red/yellow (dark/light gray) colored regions were significant in cluster/t_max_ permutation testing, respectively, suggesting information was extracted from these parts of the video (but see main text for caveat). Transparent red (gray) regions denote non-significant clusters. In the bottom part of the figure, three frames have been selected and magnified to illustrate the loss and re-emergence of cluster significance.

Illustrative results from the inferential analysis applied to the spatiotemporal experiment are shown in Figure 6. Results are shown for the ground strokes session, but were qualitatively similar for the session in which participants responded to serves. The classification video appears to reveal a spatiotemporal cluster located in the vicinity of the point of ball contact, which spans the entire time course of the video (excluding the first 25 frames, where no bubbles were applied for this experiment). However, cluster tests were applied at the level of the individual frame, rather than the entire video, and thresholding on this basis yields significant clusters in frames that form two temporally contiguous regions, the first from frame 27 to frame 85 (so around −0.6 to −0.1 s relative to racquet-ball contact) and the second from frame 95 (or 91 by t_max_ test) to frame 105. The latter region appears highly consistent with the results from the temporal and spatial sessions, suggesting information accrual from the trajectory of the ball and/or racquet head starting around the time the ball is struck.

The earlier cluster in Figure 6 is puzzling, because this region of the video should have contained no useful information to inform guesses about the subsequent shot's direction. The ground-stroke experiment was particularly revealing in this regard, because the player never occupied the region that is being marked as significant until much later on. Hence the result appears to be an artifact of some kind. We see three possibilities. First, this may simply be a false positive. However, we believe that our procedures against inflating familywise error were robust, and a similar region emerged in both ground-stroke and service sessions.

Secondly, our videos may have contained subtle differences that we failed to note, which, given that each video was presented several times, observant participants might have learnt in order to aid their discriminations. We cannot rule this out, as we did not attempt any formal investigation of potential information in this region via an ideal-observer approach. However, the earlier region of the video highlighted in Figure 6 mostly covers a blue background which was largely uniform and thus unlikely to have contained useful cues (except for chance differences in ball trajectory shortly *before* ball contact, which are visible here toward the end of the relevant period and might perhaps have been memorized across experiments).

This region is, however, remarkably consistent, spatially, with the later-emerging region that appears (based on the preceding analysis of our spatial and temporal experiments) to be a genuine locus of information accrual. Hence we suggest that the earlier region of significance may reflect an artifact caused by spatiotemporal bubbles sometimes acting as an *exogenous attentional cue* (Posner, 1980). A bubble occurring in this area of the video early during presentation would have revealed little useful information, but might, as a spatially localized transient event, have grabbed a participant's attention. On trials when a *subsequent* bubble at the same location then revealed useful information, attention would already be at this spatial location in order to assist with information extraction, thus increasing the likelihood of a correct response. Alternatively, or additionally, the earlier bubbles might not only be pointing the attentional spotlight to a relevant location, but also providing a visual predictive context for what comes next, potentially making it easier to utilize the information that was subsequently revealed in this location.

### Simulations to illustrate the impact of spatiotemporal smear

We have noted in previous sub-sections of the results that the informative regions suggested by a classification array should be treated with some caution, i.e., as containing, but potentially exaggerating in scale, regions of a video that contain information utilized by decision makers. Formally, we might consider the classification array a convolution of information-carrying regions with a filter. The properties of this filter reflect the spatiotemporal extent of the bubbles used to mask the video. While this idea is familiar to bubbles aficionados, having received discussion from the outset in the bubbles literature, it is likely less obvious to potential users from other fields. Hence, to illustrate this idea, we ran a set of simulated experiments and analyses, focussing on temporal and spatial (rather than spatiotemporal) experimental procedures (as these appear more likely to yield artifact-free results). In one set of simulations, all useful information was assumed to be contained in a single frame (temporally) or pixel (spatially). Observers' behavior (i.e., their chance of guessing correctly) was modeled as a cumulative Gaussian psychometric function of image visibility (i.e., the Bubbles profile) at the critical point, *p*, in time or space. This function was assumed to asymptote at 90% correct (as per our experimental design):

(3) $$\begin{array}{r} \text{Pr}\left(“{\text{Correct}}^{\prime \prime }\right)=0.5+0.4.\Phi \left(\frac{{\text{Bubbles}}_{p}-\mu }{\sigma_{PF}}\right) \end{array}$$

Where ϕ denotes the Standard Normal cumulative density function with mean μ and standard deviation σ_*PF*_.

**Figure 7:**
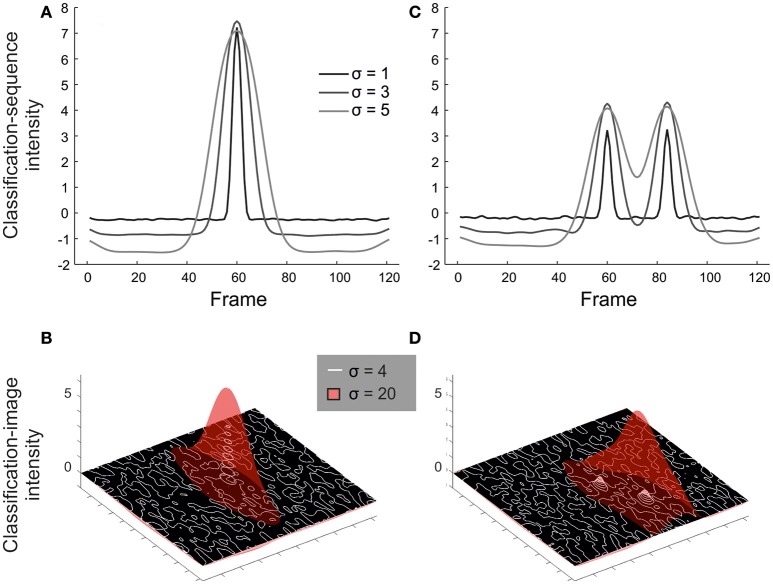
Results from illustrative simulations showing how the choice of bubble size affects the resulting classification array. Results are shown for simulations where information comes from a single frame/pixel **(A,B)** or must be seen at both of two frames/pixels **(C,D)**. The width of bubbles was varied in units of frames (**A, C:** 1 vs. 3 vs. 5) or pixels (**B, D:** 4 vs. 20). Smaller bubbles offer greater resolution for isolating small sources of information, but lack power (see especially part **D**) when information must be accrued across larger spatiotemporal scales.

Mean simulated data are presented in Figure 7A (temporal simulations) and Figure 7B (spatial simulations), varying the width of bubbles for observers modeled by a single arbitrarily selected psychometric function (σ_*PF*_ = 0.1, μ = 0.2; the pattern of results would be similar for other choices of these parameters). Notice how the resulting classification arrays are always spread out relative to the (point) information source, but even more so for bubbles with a larger width.

From the left-hand panels of Figure 7, a reasonable conclusion would be that we should use many small bubbles rather than few large bubbles, at least to the extent that the Bubbles profile can still be calculated within a reasonable period of time during an experiment. However, this is based on the assumption of a single point source informing a decision. In reality, information at various scales may prove informative. Hence we ran a second set of simulations, in which performance was modeled as a function of seeing *both* of two points of information, *p*_1_ and *p*_2_, separated by 24 frames (temporal) or ~71 pixels (spatial):

(4) $$\begin{array}{r} \text{Pr}\left(“{\text{Correct}}^{\prime \prime }\right)=0.5+0.4.\Phi \left(\frac{{\text{Bubbles}}_{p_{1}}-\mu }{\sigma_{PF}}\right).\Phi \left(\frac{{\text{Bubbles}}_{p_{2}}-\mu }{\sigma_{PF}}\right) \end{array}$$

This approximates situations in which the start and end of a larger contiguous region must be perceived to support accurate responding. Results are shown in Figures 7C,D. In cases like this, small bubbles, while precise, may reduce the magnitude of the mean classification array (and thus power to detect larger regions of information) relative to large bubbles. We would expect this difference to be exaggerated further if information from an entire contiguous region were critical.

## Discussion

Here, we set out to evaluate whether the bubbles variant of classification-image analysis (Gosselin and Schyns, 2001) could be an effective and practical tool for revealing the information extracted from real-world video stimuli to inform a speeded discrimination. We used predictions about tennis-shot direction for both forehand ground strokes and serves as a test case, bubbling our video stimuli either temporally, spatially, or spatiotemporally in a series of experiments. The results from the temporal and spatial bubbles experiments are extremely promising—the regions that emerged were consistent with the use of ball trajectory information immediately after racquet-ball contact, just as one might expect.

Our results demonstrate that the bubbles technique generalizes successfully from tightly controlled psychophysical stimuli (e.g., Gosselin and Schyns, 2001; Fiset et al., 2009; Smith et al., 2017) to videos of real-world decision-making scenarios. Although we tested just two closely related scenarios here (tennis serves and forehand ground strokes) it seems likely that the method could be further generalized. The most obvious application would be other sports, as a complement to traditional temporal and spatial occlusion paradigms. Although we did not see the anticipated differences between our novice and tennis-playing participants (for example use of kinematic information from the opponent's body by tennis players, c.f. Jackson and Mogan, 2007) this may simply reflect the nature of our tennis-playing sample, which was non-elite. It is also possible to envisage a range of other applications (e.g., in driving, and law-enforcement or military scenarios) where information extraction might helpfully be assessed. However, the results from the spatiotemporal experiment were cautionary, suggesting that this particular variant of the bubbles technique may introduce an exogenous attentional cuing artifact (c.f. Posner, 1980) that can undermine interpretation of the resulting classification videos (although other interpretations of our result cannot be ruled out). Based on the data presented here, we tentatively recommend the use of only temporal and spatial bubbles in order to avoid artifactual inferences. We speculate that by revealing regions where information is being extracted, in combination with expert knowledge about additional cues which are not being utilized, techniques like this could help inform bespoke training regimens in the future.

The strengths and limitations of bubbles need to be considered carefully when any new application is being planned. Relative to traditional spatial occlusion, the demands of stimulus preparation (i.e., frame by frame video manipulation) are reduced by a stochastic methodology. However, the bubbles method is correspondingly more complex, so the front-end investment may not be worthwhile unless a lab plans to test a range of scenarios across several experiments. We have highlighted some other considerations, for example the spatiotemporal scale of the bubbles. Small bubbles reveal information sources with high acuity, but may lack power to detect spatially or temporally extended cues. We have investigated only a single bubble size here, but some variation and/or combination of bubble sizes within a single experiment may prove more optimal when the scale of relevant information sources is hard to predict. Several ideas along these lines can be gleaned from previous work employing the bubbles technique (Chauvin et al., 2005; Blais et al., 2012).

Our work here points to a possible attention-cuing artifact for spatiotemporal bubbles, albeit one that requires further verification. However, such an artifact would really be an extreme version of a general limitation with any masking approach, which is that the masking might itself influence an observer's strategy (or their automatic processing of information) by making the image unnatural. It remains to be seen whether other forms of masking (e.g., the additive noise used in reverse correlation) could prove less disruptive in the spatiotemporal case. Clearly, tennis players do not in general see the world through bubbles, and may adapt substantially when faced with this situation. While the possible cuing artifact in our spatiotemporal experiments appears particularly egregious, it should be borne in mind that any information source revealed by bubbles reflects performance only during a bubbles experiment, not during natural viewing. For example, consider the use of information from the head/gaze, found here when predicting the direction of forehand returns. Clearly our participants *can* use this information, but it is unclear whether they would do so if bubbles did not interfere with other sources, such as ball trajectory. In general, triangulation with other complementary methodologies to assess information use (e.g., eye-tracking techniques) would be desirable, as any single technique will face interpretative limitations.

To conclude—we have demonstrated that a combination of spatial and temporal bubbles in separate experiments can be used to determine the sources of information that guide correct decisions during the real-world scenario of tennis-shot anticipation. We recommend this approach more generally, as it does not require that experimenters are required to intuit potential sources of information in advance or deliberately manipulate videos in accord with these hunches. Although initially challenging, the technique is easily adapted once it has been implemented, and has potential for much wider application within psychological and human-factors research.

## Author contributions

KY and JS conceived the experiments. SJ coded the experiments and analyses. SM ran the experiments. KY drafted the manuscript. SJ, SM, CM, JS, and KY contributed to the research design and critically revised the manuscript.

### Conflict of interest statement

The authors declare that the research was conducted in the absence of any commercial or financial relationships that could be construed as a potential conflict of interest.

## Footnotes

^1^One participant failed to provide this information.

^2^We viewed this systematic confound as acceptable, as we intended to assess the broad viability and compatibility of each approach, rather than make a detailed comparison between them, but we recognise that this choice was not ideal.

^3^To speed calculations, each bubble was rounded to zero beyond 4 (temporal) or 3 (spatial and spatiotemporal) σ from its centre. We selected a larger temporal bubble width in spatiotemporal compared to temporal sessions because a larger value allowed us to utilise fewer bubbles, and this proved important in terms of the time taken to generate each trial of the experiment.

^4^Motion in each video was detected via algorithm, and the estimated regions were then expanded slightly to ensure that no body motion was missed.

^5^In principal, this reframing can maximise power to detect information accrual at multiple points of interest in a series of analyses, but here we present data from a single coordinate transform for a relatively simple demonstration. We did explore a body-centred frame (using the navel) but it did not reveal additional sources of information missed by the analysis we present here.

^6^“4-connected” is a term from image processing and describes the manner in which connectivity is determined in a 2D or 3D space. Four-connected pixels are considered neighbours to (i.e. connected with) pixels that share a side, but not pixels that share only a corner.

^7^One typical approach to clustering in 3D data would be to use 3D connectivity to establish 3D clusters. Here, we instead used 2D connectivity *per frame* to establish 2D clusters for each frame of the video. Because we retained only the largest such cluster from the entire video for our null distribution, our 3D cluster test is, strictly, a 2D cluster test that has itself been t_max_ corrected for multiple frames.

^8^We also found no differences between these groups for serves, or in our spatial and spatiotemporal experiments, but do not illustrate all null results in order to maintain a focussed presentation.

## Supplementary material

Video S1 Video example of bubbled trials from the temporal experiment. Frame rates have been slowed to 1/4th actual presentation rate for clarity.

Video S2 Video example of bubbled trials from the spatial experiment. Frame rates have been slowed to 1/4th actual presentation rate for clarity.

Video S3 Video example of bubbled trials from the spatiotemporal experiment. Frame rates have been slowed to 1/4th actual presentation rate for clarity.

## References

[B1] Abbey C. K. Eckstein M. P. Bochud F. O. (1999). Estimation of human-observer templates in two-alternative forced-choice experiments. Proc. SPIE 3663, 284–295. 10.1117/12.349653

[B2] Abernethy B. (1988). The effects of age and expertise upon perceptual skill development in a racquet sport. Res. Q. Exerc. Sport 59, 210–221. 10.1080/02701367.1988.10605506

[B3] Abernethy B. Russell D. G. (1984). Advance cue utilisation by skilled cricket batsmen. Aust. J. Sci. Med. Sport, 16, 2–10.

[B4] Ahumada A. J. Jr. (2002). Classification image weights and internal noise level estimation. J. Vis. 2, 121–131. 10.1167/2.1.8 12678600

[B5] Ahumada A. Jr. Lovell J. (1971). Stimulus features in signal detection. J. Acoust. Soc. Am. 49, 1751–1756. 10.1121/1.1912577

[B6] Blair R. C. Karniski W. (1993). An alternative method for significance testing of waveform difference potentials. Psychophysiology 30, 518–524. 10.1111/j.1469-8986.1993.tb02075.x 8416078

[B7] Blais C. Arguin M. Gosselin F. (2013). Human visual processing oscillates: evidence from a classification image technique. Cognition 128, 353–362. 10.1016/j.cognition.2013.04.009 23764998

[B8] Blais C. Roy C. Fiset D. Arguin M. Gosselin F. (2012). The eyes are not the window to basic emotions. Neuropsychologia 50, 2830–2838. 10.1016/j.neuropsychologia.2012.08.010 22974675

[B9] Brainard D. H. (1997). The psychophysics toolbox. Spat. Vis. 10, 433–436. 10.1163/156856897X00357 9176952

[B10] Butler S. Blais C. Gosselin F. Bub D. Fiset D. (2010). Recognizing famous people. Attention Percept. Psychophys. 72, 1444–1449. 10.3758/APP.72.6.1444 20675791

[B11] Chauvin A. Worsley K. J. Schyns P. G. Arguin M. Gosselin F. (2005). Accurate statistical tests for smooth classification images. J. Vis. 5, 659–667. 10.1167/5.9.1 16356076

[B12] Farrow D. Abernethy B. Jackson R. C. (2005). Probing expert anticipation with the temporal occlusion paradigm: experimental investigations of some methodological issues. Motor Control 9, 330–349. 10.1123/mcj.9.3.330 16239719

[B13] Fiset D. Blais C. Arguin M. Tadros K. Ethier-Majcher C. Bub D.. (2009). The spatio-temporal dynamics of visual letter recognition. Cogn. Neuropsychol. 26, 23–35. 10.1080/02643290802421160 18979274

[B14] Gosselin F. Schyns P. G. (2001). Bubbles: a technique to reveal the use of information in recognition tasks. Vision Res. 41, 2261–2271. 10.1016/S0042-6989(01)00097-9 11448718

[B15] Graham N. V. S. (1989). Visual Pattern Analyzers. New York, NY: Oxford University Press.

[B16] Groppe D. M. Urbach T. P. Kutas M. (2011). Mass univariate analysis of event-related brain potentials/fields I: a critical tutorial review. Psychophysiology 48, 1711–1725. 10.1111/j.1469-8986.2011.01273.x 21895683 PMC4060794

[B17] Jackson R. C. Mogan P. (2007). Advance visual information, awareness, and anticipation skill. J. Mot. Behav. 39, 341–351. 10.3200/JMBR.39.5.341-352 17827112

[B18] Jones C. Miles T. (1978). Use of advance cues in predicting the flight of a lawn tennis ball. J. Hum. Mov. Stud. 4, 231–235.

[B19] Kleiner M. Brainard D. Pelli D. Ingling A. Murray R. Broussard C. (2007). What's new in psychtoolbox-3. Perception 36:1.

[B20] Marmarelis P. Z. Naka K. (1972). White-noise analysis of a neuron chain: an application of the wiener theory. Science 175, 1276–1278. 10.1126/science.175.4027.1276 5061252

[B21] Muller S. Abernethy B. Farrow D. (2006). How do world-class cricket batsmen anticipate a bowler's intention? Quart. J. Exp. Psychol. 59, 2162–2186. 10.1080/02643290600576595 17095494

[B22] Nichols T. E. Holmes A. P. (2002). Nonparametric permutation tests for functional neuroimaging: a primer with examples. Hum. Brain Mapp. 15, 1–25. 10.1002/hbm.1058 11747097 PMC6871862

[B23] Pelli D. G. (1997). The videotoolbox software for visual psychophysics: transforming numbers into movies. Spat. Vis. 10, 437–442. 10.1163/156856897X00366 9176953

[B24] Posner M. I. (1980). Orienting of attention. Q. J. Exp. Psychol. 32, 3–25. 10.1080/00335558008248231 7367577

[B25] Shim J. Carlton L. G. Kwon Y. (2006). Perception of kinematic characteristics of tennis strokes for anticipating stroke type and direction. Res. Q. Exerc. Sport 77, 326–339. 10.1080/02701367.2006.10599367 17020077

[B26] Simoncelli E. P. Paninski L. Pillow J. Schwartz O. (2004). Characterization of neural responses with stochastic stimuli, in The New Cognitive Neurosciences, 3rd edn., ed M. Gazzaniga (Cambridge, MA: MIT Press), 327–338.

[B27] Smith M. L. Cesana M. L. Farran E. K. Karmiloff-Smith A. Ewing L. (2017). A “spoon full of sugar” helps the medicine go down: how a participant friendly version of a psychophysics task significantly improves task engagement, performance and data quality in a typical adult sample. Behav. Res. Methods 50, 1011–1019. 10.3758/s13428-017-0922-6 28646402

[B28] Thurman S. M. Grossman E. D. (2008). Temporal “Bubbles” reveal key features for point-light biological motion perception. J. Vis. 8, 28–28. 10.1167/8.3.28 18484834

[B29] Vinette C. Gosselin F. Schyns P. G. (2004). Spatio-temporal dynamics of face recognition in a flash: it's in the eyes. Cogn. Sci. 28, 289–301. 10.1016/j.cogsci.2004.01.002

[B30] Watson A. B. Pelli D. G. (1983). QUEST: a bayesian adaptive psychometric method. Attention Percept. Psychophys. 33, 113–120. 10.3758/BF03202828 6844102

[B31] Yarrow K. Brown P. Krakauer J. W. (2009). Inside the brain of an elite athlete: the neural processes that support high achievement in sports. Nat. Rev. Neurosci. 10, 585–596. 10.1038/nrn2672 19571792

